
JOURNAL INFORMATION
==============================
NLM Title Abbreviation: Wirtschaftsdienst
Journal ID: Wirtschaftsdienst
NLM Journal ID: 101086612

Wirtschaftsdienst (Hamburg, Germany : 1949)

ISSN: 0043-6275

ARTICLE INFORMATION
==============================
PMCID: PMC7441474
PMID: 32843776
DOI: 10.1007/s10273-020-2725-0
Article ID: 2725
Article version: 1
Subjects: Ökonomische Trends

Der Einfluss von COVID-19 auf die Rohstoffmärkte

The influence of COVID-19 on the commodity markets

Wellenreuther Claudia wellenreuther@hwwi.org

grid.435054.3 0000 0001 2227 8589 HWWI, Oberhafenstraße 1, 20097 Hamburg, Deutschland
Electronic publication date: 2020 Aug 21
Print publication date: 2020
Volume: 100
Issue: 8
First page: 643
Last page: 644
Copyright: © Der/die Autor(en) 2020
License: Open Access Dieser Artikel wird unter der Creative Commons Namensnennung 4.0 International Lizenz veröffentlicht, welche die Nutzung, Vervielfältigung, Bearbeitung, Verbreitung und Wiedergabe in jeglichem Medium und Format erlaubt, sofern Sie den/die ursprünglichen Autor(en) und die Quelle ordnungsgemäß nennen, einen Link zur Creative Commons Lizenz beifügen und angeben, ob Änderungen vorgenommen wurden.
Die in diesem Artikel enthaltenen Bilder und sonstiges Drittmaterial unterliegen ebenfalls der genannten Creative Commons Lizenz, sofern sich aus der Abbildungslegende nichts anderes ergibt. Sofern das betreffende Material nicht unter der genannten Creative Commons Lizenz steht und die betreffende Handlung nicht nach gesetzlichen Vorschriften erlaubt ist, ist für die oben aufgeführten Weiterverwendungen des Materials die Einwilligung des jeweiligen Rechteinhabers einzuholen.
Weitere Details zur Lizenz entnehmen Sie bitte der Lizenzinformation auf http://creativecommons.org/licenses/by/4.0/deed.de.
License URL: https://creativecommons.org/licenses/by/4.0/

issue-copyright-statement: © The Author(s) 2020

==============================

Die globale Ausbreitung des neuartigen Coronavirus im ersten Halbjahr 2020 wurde von einem deutlichen Preisverfall auf den internationalen Rohstoffmärkten begleitet. Die weltweit verhängten Lockdown-Maßnahmen zur Eindämmung der durch das Coronavirus ausgelösten Pandemie bremsten die globale Wirtschaft und prägten die Entwicklung auf den internationalen Rohstoffmärkten maßgeblich. Die Lockdown-Maßnahmen wirkten durch verschiedene Kanäle sowohl auf die Nachfrage als auch auf das Angebot von Rohstoffen und beeinflussten dabei die Preise nahezu aller Rohstoffe aus den unterschiedlichen Segmenten. Die Rohölpreise wurden insbesondere durch eine geringere Treibstoffnachfrage aufgrund von strengen Beschränkungen des Reise- und Transportverkehrs beeinflusst. Die gebremste globale wirtschaftliche Aktivität reduzierte ebenfalls die Rohölnachfrage und zusätzlich in großem Maße die Nachfrage nach Industrierohstoffen. Der Preisverfall auf den Industrie- und Agrarrohstoffmärkten wurde jedoch durch eine Verknappung des Angebots aufgrund von Störungen der Lieferketten und der Produktion abgeschwächt (World Bank Group, 2020). Im ersten Quartal 2020 sank der HWWI-Rohstoffpreisindex, der die preislichen Veränderungen der 31 wichtigsten internationalen Rohstoffe aus den Bereichen Energie, Nahrungs- und Genussmittel sowie Industrierohstoffe abbildet, um 17 % im Vergleich zum Vorquartal und um 20 % verglichen mit dem ersten Quartal 2019. Im zweiten Quartal 2020 fiel der HWWI-Rohstoffpreisindex um weitere 35 % und im Vorjahresvergleich um 49 %. Der stärkste Preisverfall auf den Rohstoffmärkten konnte im April 2020 beobachtet werden (vgl. Abbildung 1).

Die weltweit verhängten Maßnahmen zur Bekämpfung der Corona-Pandemie trafen die Rohölmärkte am stärksten. Zunächst in China und danach weltweit wurden Ausgangsbeschränkungen verhängt, die zu leeren Straßen, einem starken Rückgang des Flugverkehrs und einer heruntergefahrenen Industrie führten. Besonders stark fiel der Nachfragerückgang aus dem Transportsektor ins Gewicht, da dieser fast zwei Drittel der gesamten Rohölnachfrage ausmacht (World Bank Group, 2020). Nach Zahlen der U.S. Energy Information Administration (2020) erreichte der weltweite Verbrauch an Rohöl im April 2020 historische Tiefstwerte von durchschnittlich 79 Mio. Barrel pro Tag, was einem Nachfragerückgang von 21 Mio. Barrel pro Tag im Vergleich zum April des Vorjahres entsprach. Der Einbruch der globalen Rohölnachfrage spiegelte sich in einem enormen Preisverfall wider. So lag der durchschnittliche Preis der europäischen Referenzsorte Brent im April unter 30 US-$ pro Barrel und damit über 60 % unter dem durchschnittlichen Preis im April 2019 (vgl. Abbildung 2).[1] Die rückläufige Preisentwicklung wurde zusätzlich dadurch verstärkt, dass sich die OPEC+-Staaten[2], insbesondere Saudi-Arabien und Russland, im März nicht auf Förderungskürzungen einigen konnten, um den Ölpreis zu stützen. Die ergebnislosen Verhandlungen führten zu einem Preiskrieg zwischen Saudi-Arabien und Russland und zu einer Ausweitung des Angebots an Rohöl. Erst Ende April 2020 konnte eine Einigung über Förderungskürzungen erzielt werden (OPEC, 2020). Im Mai und Juni verringerten die OPEC+-Staaten ihr Angebot um 9,7 Mio. Barrel pro Tag, was einer weltweiten Angebotskürzung von etwa 10 % entsprach. Zusätzlich zu den Förde-

1 Im April verzeichnete der Preis für die amerikanische Referenzsorte WTI erstmalig negative Werte. Der Futures-Preis für den Mai-Kontrakt für ein Barrel WTI Rohöl rutschte am 20. April 2020 auf -37,63 US-$. Der Grund für den kurzfristig negativen Preis war das Auslaufen des Mai-Terminkontrakts. Händler wollten ihre Kaufposition vor Fristende verkaufen, um einer physischen Lieferung zu entgehen und hatten Schwierigkeiten, Käufer für ihre Positionen zu finden.

2 Die Organisation der erdölexportierenden Länder (OPEC) umfasst zurzeit die folgenden Mitglieder: Algerien, Angola, Ecuador, Äquatorialguinea, Gabun, Iran, Irak, die Republik Kongo, Kuwait, Libyen, Nigeria, Saudi-Arabien, die Vereinigten Arabischen Emirate und Venezuela. OPEC+ umschließt noch weitere Förderländer inklusive Russland.

Abbildung 1
**HWWI-Rohstoffpreisindex**
Index auf US-$-Basis, 2015 = 100

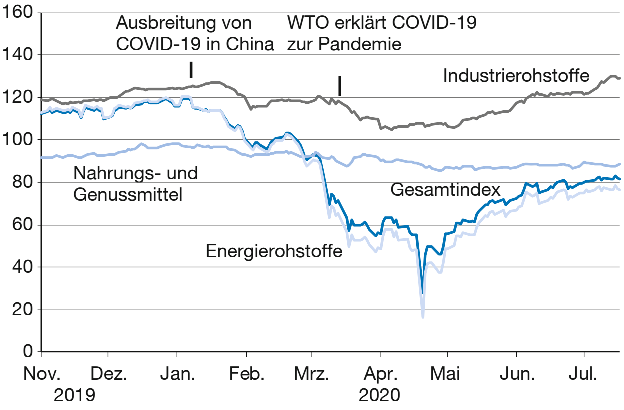

Quelle: Hamburgisches WeltWirtschaftsInstitut (HWWI).

Abbildung 2
**Entwicklungen auf dem Rohölmarkt**

rungskürzungen der OPEC+ ist die Schieferölproduktion der USA eingebrochen, da aufgrund der stark gesunkenen Rohölpreise amerikanische Schieferölproduzenten gezwungen waren, unrentable Bohrlöcher zu schließen. Zusammen mit einer langsamen Zunahme der Nachfrage nach Rohöl, begründet durch die Erholung der chinesischen Wirtschaft und der sukzessiven Lockerung der Lockdown-Maßnahmen in vielen weiteren Ländern sind seit Mai wieder steigende Rohölpreise zu beobachten.

Die Preise für Industrierohstoffe fielen im ersten Quartal des Jahres ebenfalls, jedoch in einem geringeren Ausmaß als die Preise für Rohöl. Im Gegensatz zu den Rohölmärkten reduzierte der weltweite Shutdown zur Eindämmung des Coronavirus neben der Nachfrage auch das Angebot von Industriemetallen. Die Entwicklung der Preise für Industriemetalle steht in einem engen Zusammenhang zu der Entwicklung der globalen Wirtschaft. Insbesondere die chinesische Industrie ist hier von großer Bedeutung, da China weltweit nicht nur größter Produzent, sondern auch größter Konsument von Industriemetallen ist. Der Lockdown in China sorgte bereits im Februar für einen Nachfragerückgang und damit für Preiseinbrüche, insbesondere auf den Märkten für Zink und Kupfer. Abgeschwächt wurde der Preisverfall durch Verknappungen des Angebots aufgrund der Lockdown-Maßnahmen, die zeitweise zu Schließungen von Minen und Raffinerien führten. Seit April macht sich die schnelle Erholung der chinesischen Industrieproduktion in steigenden Industriemetallpreisen bemerkbar.

Auf den Märkten für Agrarrohstoffe waren gegenläufige Entwicklungen zu beobachten. Im März und April 2020 wiesen die Preise für Zucker, Mais und Pflanzenöle einen deutlichen Rückgang auf, da die bedingt durch die Corona-Krise weltweit eingebrochene Nachfrage nach Treibstoffen auch zu einem Einbruch der Biotreibstoffproduktion führte. Der Stillstand der Autoindustrie führte zu einem Nachfragerückgang an Kautschuk, das für die Produktion von Autoreifen verwendet wird. Gegensätzlich verhielten sich die Preise für Grundnahrungsmittel wie Gerste, Weizen und Reis. Hier wirkte sich die Corona-Krise in einen Anstieg der Nachfrage bei gleichzeitiger Verknappung des Angebots aus und führte zu Preisanstiegen. Die Nachfrage stieg deutlich an, da viele Länder vermehrt Grundnahrungsmittel kauften, um möglichen Lebensmittelknappheiten aufgrund der Corona-Krise entgegenzuwirken. Der Reispreis verzeichnete einen besonders starken Anstieg, da viele asiatische Produzentenländer, unter anderem Indien, landesweite Ausgangssperren anordneten, um die Ausbreitung des Coronavirus zu verhindern. Die strengen Ausgangssperren führten zu Problemen bei der Reisernte aufgrund von Arbeitskräftemangel und logistischen Schwierigkeiten. Zusätzlich dazu stoppten Produzentenländer wie Indien, Vietnam und Thailand ihre Reisexporte, um die Lebensmittelversorgung im eigenen Land zu gewährleisten. Ebenfalls aus Angst vor Nahrungsmittelknappheit lagerten Bauern und Händler verstärkt Grundnahrungsmittel ein und Konsumenten erhöhten ihre Einkäufe.

Die Entwicklungen auf den internationalen Rohstoffmärkten waren im ersten Halbjahr 2020 somit maßgeblich durch die Corona-Pandemie geprägt. Die Lockdown-Maßnahmen zur Bekämpfung der Corona-Pandemie lösten eine Kombination aus Angebots- und Nachfrageschock auf den Rohstoffmärkten aus. Durch die rasche Erholung der chinesischen Wirtschaft und auch durch die Lockerung der weltweiten Lockdown-Maßnahmen sind seit Mai Nachfrageanstiege und steigende Preise auf den internationalen Rohstoffmärkten zu beobachten. Wie sich die Rohstoffpreise zukünftig entwickeln werden, hängt stark von der Entwicklung der Corona-Pandemie und den jeweiligen Reaktionen der einzelnen Länder ab. Eine zweite Welle und weitere Lockdown-Maßnahmen, insbesondere in China, könnten die Rohstoffmärkte erneut stark beeinflussen.

Claudia Wellenreuther
wellenreuther@hwwi.org


Literatur

OPEC (2020), The 10th (Extraordinary) OPEC and non-OPEC Ministerial Meeting concludes, 12 April, https://www.opec.org/opec_web/en/press_room/5891.htm, (31. Juli 2020).
U.S. Energy Information Administration (2020), Short-Term Energy Outlook, https://www.eia.gov/outlooks/steo/pdf/steo_full.pdf (31. Juli 2020).
World Bank Group (2020), Commodity Markets Outlook, April, https://openknowledge.worldbank.org/handle/10986/33624 (31. Juli 2020).
